# Successful Treatment of Rotavirus-induced Diarrhoea in Suckling Mice with Egg Yolk Immunoglobulin

**Published:** 2007-12

**Authors:** Shafiqul A. Sarker, Neha Pant, Lekh R. Juneja, Lennart Hammarström

**Affiliations:** 1 ICDDR,B, GPO Box 128, Dhaka 1000, Bangladesh; 2 Karolinska University Hospital, Huddinge, Stockholm, Sweden; 3 Nutritional Foods Division, Taiyo Kagaku Co. Ltd., Akahoiri Shinmachi, Yokkaichi, Japan

**Keywords:** Clinical trials, Diarrhoea, Disease model, Animal, Immunoglobulins, Rotavirus

## Abstract

The role of specific immunoglobulins at mucosal sites in imparting protection against disease, such as rotavirus-associated diarrhoea, is well-established. Oral immunoglobulin therapy with egg yolk-derived antirotavirus immunoglobulins has previously been shown to achieve moderate therapeutic effect in diarrhoea due to rotavirus in a clinical trial. Here, data on the therapeutic potential of the same immunoglobulin preparation in an infant mouse model of rotavirus-induced diarrhoea is presented. The use of an animal model has allowed therapy to be evaluated with higher doses of immunoglobulins and has suggested that an improved therapeutic effect can be achieved by increasing the dose in the clinical setting.

## INTRODUCTION

Rotavirus, the leading aetiological agent of severe gastroenteritis in infants and young children, is an important public-health problem worldwide. It infects about 90% of humans before three years of age, causing an estimated 125 million episodes of diarrhoea annually in children aged less than five years in developing countries (1). The high mortality and morbidity associated with rotavirus-associated gastroenteritis makes its treatment and prevention an important public-health goal. However, specific, effective, and affordable therapy is currently not available. Two rotavirus vaccines—Rotarix (Glaxo Smith Kline Biologicals, Belgium) and RotaTeq (Merck & Co., USA)—have recently completed Phase III clinical trials. Both the vaccines were efficacious in preventing severe gastroenteritis, predominantly due to the G1 rotavirus serotype (2). However, their use is limited to children aged less than three months, and confirmation of their safety will require extensive post-marketing surveys and continuous epidemiological surveillance on circulating strains will be required wherever the vaccines are introduced. There is, thus, a clear need to define improved, cost-effective interventions in the management of diarrhoea due to rotavirus until the time an effective, safe, and inexpensive vaccine, affordable to developing countries, is available.

Oral administration of virus-neutralizing specific antibodies is an attractive approach for passive protection of humans and animals against gastrointestinal pathogens. We have documented the successful treatment of rotavirus-associated diarrhoea in children with immunoglobulin from immunized cow colostrums with titres of 7,500 against serotype 1, 2,000 against serotype 2, 4,500 against serotype 3, and 4,500 against serotype 4 (3). In laying hens, immunoglobulin is transported to the yolk of eggs and provides passive immunity to the chicks. Eggs from hens immunized against different antigens are, thus, a rich source for harvesting specific immunoglobulins (IgY). Purified IgY has previously been produced and used against a number of mucosal pathogens, including rotavirus. In a clinical trial by Sarker and colleagues that evaluated therapy of severe diarrhoea due to rotavirus using specific IgY, a modest beneficial effect of the treatment in reducing stool output and achieving faster clearance from virus was observed (4). However, the main clinical outcome of disease, i.e. duration of diarrhoea, remained unchanged. Protection achieved by passive immunotherapy does not involve active participation of the host's immune system but reflects solely the effect of extraneously-delivered immunoglobulins. For a successful passive immunotherapy, there has to be enough immunoglobulins present at the mucosal site to control the infection process, a premise that is also supported in the observed dose dependency of passive immunotherapy in controlling infections (5).

Using immunoglobulins prophylactically results in an improved outcome since the infection can be easily stifled. However, prophylactic administration of immunoglobulins is usually not possible. To our knowledge, the clinical trial by Sarker and colleagues (4) was the first one and only therapeutic evaluation of IgY against rotavirus in humans. The titre of IgY in the final preparation was relatively low, and only 1.5 g of immunoglobulins (in 10 g of immunized egg yolk powder) could be given per day. Although the therapeutic efficacy of IgY in this trial was modest, using a small animal model of rotavirus-associated diarrhoea, we investigated whether an improved therapeutic effect could be achieved using higher doses of IgY.

## MATERIALS AND METHODS

### Anti-rotavirus IgY immunoglobulins

The egg yolk immunoglobulin was obtained from Nutritional Foods Division, Taiyo Kagaku Co., Ltd., Yokkaichi, Japan. The production of hyperimmunized egg yolk (HEY) immunoglobulin has been described previously by Hatta *et al.* (6,7). In brief, white leghorn hens, aged 150 days, were immunized intramuscularly with 1×10^7^ fluorescent cell-forming units (FCFUs) of human rotavirus strains—Wa, RV5, RV3, and ST3—twice at an interval of one week using Freund's complete adjuvant. After the initial immunization, eggs were collected daily and stored in the cold temperature for two weeks. Then, their yolks were separated, pooled, and frozen for purification of IgY. The purification of HEY from pooled eggs, collected 4 to 22 weeks after immunization, was in accordance with the procedures described by Hatta *et al.* (6,7) and Polson *et al.* (8). The neutralization titre, measured in a neutralization test against serotype 3, was 1:100,000 (9). The protein content of the preparations was 68.7%, with an IgY content of 20%.

### Animal experiments

Fourteen-day pregnant, rotavirus-negative Balb/c mice were obtained from Taconic Labs, Denmark. The mice were housed individually in the animal facility at Huddinge Hospital, Stockholm, Sweden. Normal pellet diet and water was provided *ad libitum*. The Animal Ethics Committee of Karolinska University Hospital at Huddinge, Stockholm, approved the study. Pups were born on day 19-20 of gestation, and four-day old pups were randomized to different experimental groups. Pups were orally challenged with 2×10^7^ foci-forming units (FFUs) of rhesus rotavirus (strain RRV) in a 10-μl volume on day 0 of experiment. Feeding with anti-rotavirus IgY in a total volume of 10 μl phosphate-buffered saline commenced from day 1 to day 5. Four different doses of IgY were tested: 10 mg/mL (n=15), 1 mg/mL (n=15), 0.1 mg/mL (n=11), and 0.01 mg/mL (n=7). The control group (n=15) included pups that were only infected. The pups were checked daily for signs of diarrhoea, which included loose stools on gentle palpation of the abdomen or a smeared tail during the six days of the experiment. The outcome measures were prevalence of diarrhoea (defined as the percentage of pups with diarrhoea on a daily basis) and duration of diarrhoea (defined as time in days with diarrhoea).

### Statistics

The chi-squared test (Epi Info software, version 3.4, 2005) was used for comparing the proportion of pups with diarrhoea following RRV challenge and treatment with different concentrations of IgY. The duration of diarrhoea among the pups with different interventions was compared with one-way analysis of variance (ANOVA), and that between two groups was compared by the Student's t-test (SPSS software, version 10 Windows).

## RESULTS

Oral inoculation of RRV typically induced diarrhoea 24 hours post-inoculation with the disease peaking around day 3 to day 4. A positive outcome of therapeutic feeding with IgY and a clear dose dependency could be observed. Four different doses of IgY were tested from 0.01 mg/mL up to 10 mg/mL. Feeding higher doses of IgY (1 mg/mL) had a stronger effect on the prevalence of diarrhoea on day 4 (33% vs 67% for control, p=0.006). The beneficial effect was more pronounced in pups receiving the 10-mg/mL dose on day 4 (6% vs 67% for control, p=0.0006). Feeding lower doses resulted in no significant effect on the prevalence of diarrhoea (63% for 0.1 mg/mL and 86 % for 0.01 mg/mL compared to 67% for controls, p=NS) (Table 1).

**Table 1:** Therapeutic effect of different doses of orally-administered IgY against RRV-induced diarrhoea in infant mice

	Day 1	Day 2	Day 3	Day 4
Treatment group	No. (%) with diarrhoea	No. (%) with diarrhoea	No. (%) with diarrhoea	No. (%) with diarrhoea

IgY 10 mg/mL (n=15)	3 (20)	5 (33)	4 (27)	1 (6)
IgY 1 mg/mL (n=15)	4 (27)	7 (47)	6 (40)	5 (33)
IgY 0.1 mg/mL (n=11)	2 (18)	5 (45)	7 (63)	7 (63)
IgY 0.01 mg/mL (n=7)	0	5 (71)	3 (43)	6 (86)
RRV (control) (n=15)	8 (53)	13 (87)	12 (80)	10 (67)

IgY 10 mg/mL vs control: Day 1: χ^2^=3.6, p=0.06, RR 0.12 (95% CI 0.38-1.15); Day 2: χ^2^=8.9, p=0.003, RR 0.38 (95% CI 0.18-0.81); Day 3: χ^2^=8.6, p=0.003, RR 0.50 (95% CI 0.39-0.65); Day 4: χ^2^=11.6, p=0.0006, RR 0.10 (95% CI 0.1-0.69)				
IgY 1 mg /mL vs control: Day 1: χ^2^=2.2, p=0.13, RR 0.05 (95% CI 0.32-1.85); Day 2: χ^2^=5.4, p=0.020, RR 0.54 (95% CI 0.30-0.96); Day 3: χ^2^=5.0, p=0.025, RR 0.50 (95% CI 0.26-0.98); Day 4: χ^2^=3.3, p=0.06, RR 0.50 (95% CI 0.22-1.1)				

CI=Confidence interval; IgY=Yolk immunoglobulin; RR=Relative risk; RRV=Rhesus rotavirus

The duration of diarrhoea was also reduced with treatment with IgY and showed a dose-dependent behaviour. Lower doses with 0.01 mg/mL and 0.1 mg/mL resulted in significant reduction or slight effect on duration of diarrhoea compared to the control. On the contrary, larger doses elicited significant relative reduction in duration of diarrhoea compared to the control mice or even mice receiving lower amounts of IgY (F=6.7, p=<0.0001). Treatment with the 10-mg/mL dose achieved the strongest reduction compared to the control mice or even mice receiving lower amounts of IgY (69% vs 17% for 0.1 mg/mL, 31% for 0.01 mg/mL) (Table 2).

**Table 2:** Reduction in duration of rotavirus-induced diarrhoea in mouse pups after oral administration of different concentration of anti-rotavirus IgY∗

Treatment group	Duration of diarrhoea (days)	% of relative reduction compared to control	F value	p value
IgY 10 mg/mL (n=15)†	0.9±1.0	69		
IgY 1 mg/mL (n=15)‡	1.7±1.2	41		
IgY 0.1 mg/mL (n=11)§	2.4±1.3	17		
IgY 0.01 mg/mL (n=7)§	2.0±1.2	31	6.7	<0.0001
RRV (control) (n=15)	2.9±1.2	-		

∗One way analysis of variance (ANOVA);

†p values are significant with all groups;

‡p value is significant with control only;

§p value is not significant with control

## DISCUSSION

In this study, we have used a well-characterized standard rotavirus infection model in Balb/c mice previously described by Ruggeri and co-workers (10). Oral inoculation with RRV induces typical signs of rotavirus-associated diarrhoea with loose stools and associated histopathological changes (Pant N. Personal communication, 2006). We have recently used this animal model for testing a yeast-produced, monovalent, llama antibody fragment against rotavirus (11). Here, we have evaluated the therapeutic potential of anti-rotavirus IgY. Chicken IgY is homologous to the mammalian IgG and is associated with transferring passive immunity to the maturing egg. Hens can readily be hyperimmunized, and the concentration of IgY in the egg yolk can be as high as 25 mg/mL (12), making IgY a very attractive source of passive immunization. With reference to rotavirus, IgY has been used experimentally in mice (5,13,14) and calves (15). However, these studies have only evaluated the effect of prophylactic administration of IgY. Prophylactic administration of immunoglobulins represents an ideal situation where, owing to the presence of immunoglobulins even before the pathogen is introduced, the protection is immediate. Although administering immunoglobulins prophylactically may shed some light on their potential clinical use, it seldom represents the real-life scenario. We have, therefore, evaluated therapeutic intervention using four different doses of IgY after challenging mice with rotavirus. Results from our current study demonstrate that therapy with IgY is clearly dose-dependent with the highest dose of 10 mg/mL imparting the strongest protection at least in mouse pups. As originally designed, we have not fixed the dose according to that used in the clinical study (4) or according to body-weight. The doses used in this study were arbitrarily chosen beginning from a lower to a higher dose to test the hypothesis that an improved therapeutic effect could be achieved using higher doses of IgY in mice and then extrapolating/hypothesing that more would probably have been better to have used more in the human study as well, since we got rather poor clinical results.

We have previously conducted a randomized double-blind clinical trial testing the therapeutic ability of IgY in patients with severe diarrhoea due to rotavirus (4). In this study, we had used a standard dose of 1.5 g IgY (in 10 g of immunized egg yolk powder) per day for four days. Treatment with IgY caused a faster clearance of virus shedding but did not reduce the duration of the disease. Presumably, at the time the patients were admitted to the trial, they had the peak of infection with high levels of rotavirus particles. Based on our current results, it is likely that using higher doses of IgY, or a product with a higher titre for treatment, would have given a better outcome in the previous clinical trial and should be considered in designing such trials in future.

## References

[1] Cook SM, Glass RI, LeBaron CW, Ho M-S (1990). Global seasonality of rotavirus infections. Bull World Health Organ.

[2] Cunliffe NA, Nakagomi O (2005). A critical time for rotavirus vaccines: a review. Expert Rev Vaccines.

[3] Sarker SA, Casswall TH, Mahalanabis D, Alam NH, Albert MJ, Fuchs G (1998). Successful treatment of rotavirus diarrhoea in children with immunoglobulin from immunized bovine colostrum. Pediatr Infect Dis J.

[4] Sarker SA, Casswall TH, Juneja LR, Hoq E, Hossain I, Fuchs GJ (2001). Randomized, placebo–controlled, clinical trial of hyperimmunized chicken egg yolk immunoglobulin in children with rotavirus diarrhea. J Pediatr Gastroenterol Nutr.

[5] Ebina T, Tsukada K, Umezu K, Nose M, Tsuda K, Hatta H (1990). Gastroenteritis in suckling mice caused by human rotavirus can be prevented with egg yolk immunoglobulin (IgY) and treated with a protein-bound polysaccharide preparation (PSK). Microbial Immunol.

[6] Hatta H, Kim M, Yamamoto T (1990). A novel isolation method for hen egg yolk antibody, “IgY”. Agric Biol Chem.

[7] Hatta H, Tsuda K, Akachi S, Kim M, Yamamoto T (1993). Productivity and some properties of egg yolk antibody (IgY) against human rotavirus compared with rabbit IgG. Biosci Biotechnol Biochem.

[8] Polson A, von Wechmar MB (1980). Isolation of viral IgY antibodies from yolks of immunized hens. Immunol Commun.

[9] Hatta H, Tsada K, Ozeki M, Kim M, Yamamoto T, Otake S (1997). Passive immunization against dental plaque formation in humans: effect of a mouth rinse containing egg yolk antibodies (IgY) specific to Streptococcus mutans. Caries Res.

[10] Ruggeri FM, Johansen K, Basile G, Kraehenbuhl JP, Svensson L (1998). Antirotavirus immunoglobulin A neutralizes virus in vitro after transcytosis through epithelial cells and protects infant mice from diarrhea. J Virol.

[11] van der Vaart JM, Wolvers D, Pant N, Bezemer S, Hermans P, Bellamy K (2006). Production of monovalent llama-derived antibody directed against a common rotavirus strain and their effectiveness against rotavirus induced diarrhea in mice. Vaccine.

[12] Rose ME, Orlans E, Buttres N (1974). Immunoglobulin classes in the hens egg: their segregation in yolk and white. Eur J Immunol.

[13] Bartz CR, Conklin RH, Tunstall CB, Steele JH (1980). Prevention of murine rotavirus infection with chicken egg yolk immunoglobulins. J Infect Dis.

[14] Yolken RH, Leister F, Wee S-B, Miskuff R, Vonderfecht S (1988). Antibodies to rotaviruses in chickens’ eggs: a potential source of antiviral immunoglobulins suitable for human consumption. Pediatrics.

[15] Kuroki M, Ohta M, Ikemori Y, Peralta RC, Yokoyama H, Komada Y (1994). Passive protection against bovine rotavirus in calves by specific immunoglobulins from chicken egg yolk. Arch Virol.

